# Supplementary material for: Carbon dioxide regulates Mycobacterium tuberculosis PhoPR signaling and virulence
Source: Infect Immun. 2025 Feb 18;93(3):e00568-24. doi: 10.1128/iai.00568-24 (PMC11895460; doi:10.1128/iai.00568-24)
Supplement: Supplemental material — Table S1; Fig. S1 to S5. [file iai.00568-24-s0001.pdf]

Table S1 Plasmids and primers used in this study.

Plasmid or Primer Name	Characteristics or Sequence (5'→ 3')	Reference
pKM444	Kan[R]; Mycobacterial shuttle vector expressing the Che9c phage RecT annealase and the Bxb1 phage integrase from the P_{tet} promoter	Murphy *et. al.* (2018)
pKM464	Hyg[R]; Mycobacterial integration vector for deleting target gene, insertion of Bxb1 *attB* site from the P_{Hyg} promoter	Murphy *et. al.* (2018)
canC (ORBIT oligomer)[a]	Ggcgaacacaatgccgtgtttctggcccggccctgacgctgtgaccattccgaggagtcaacacatgagc**GGTTTGTCTGGTCAACCACCGCGGTCTCAGTGGTGTACGGTACAAACC**cgccccgtcgaccacgaatcagcgcagtagcgcccgcgacatcactacccgctgaatctgattggtgccc	This Study
oriE	cctggtatctttatagtcctgtcg	Murphy *et. al.* (2018)
HygC-out	tgcacgggaccaacaccttcgtgg OR gaggaactggcgcagttcctctgg	Murphy *et. al.* (2018)
Seq-*canC*-For	agaacgacctcaccctggaagtcg	This Study
Seq-*canC*-Rev	gaggtcaccaccgatgccgtacaa	ThisStudy
PLJR965	Kan[R]; Plasmid co-expressing $dCas9_{Sth1}$ and targeting sgRNA under the control TetR-regulated *dcas9* promoter	Rock *et. al.* (2017)
canA Pam1-FWD	gggagtgcctcgccctccttgatgc	This Study
canA Pam1-REV	aaacgcatcaaggagggcgaggcac	This Study
canA Pam2-FWD	gggagaagtcgtcgtcggtgaaag	This Study
canA Pam2-REV	aaacctttcaccgacgacgacttc	This Study
canA Pam3-FWD	gggaatcccacagtcggtgtggtgca	This Study
canA Pam3-REV	aaactgcaccacaccgactgtgggat	This Study
canB Pam1-FWD	gggagatggccgatgaacgcgcca	This Study
canB Pam1-REV	aaactggcgcgttcatcggccatc	This Study
canB Pam2-FWD	gggaactcagaccgtcacggcggc	This Study
canB Pam2-REV	aaacgccgccgtgacggtctgagt	This Study
canC Pam1-FWD	gggagtttgggcaagccaatcgcgtc	This Study
canC Pam1-REV	aaacgacgcgattggcttgcccaaac	This Study
canC Pam2-FWD	gggagctggcggtgatgacgttcg	This Study
canC Pam2-REV	aaaccgaacgtcatcaccgccagc	This Study
canC Pam3-FWD	gggaagcgaaagtggcaacgcaac	This Study
canC Pam3-REV	aaacgttgcgttgccactttcgct	This Study
RTpcr-*canA*-FWD	acgactacctggccaacaac	This Study
RTpcr-*canA*-REV	cagtgaacggatcacatcgt	This Study
RTpcr-*canB*-FWD	tgagtcgtgtcgacgagttc	This Study
RTpcr-*canB*-REV	gcccatcgtcgagttgatag	This Study
RTpcr-*canC*-FWD	ctgatccgattggactggtt	This Study
RTpcr-*canC*-REV	cacaggtgaggaacagctca	This Study

[a]The Bxb1 phage *attP* sequence

Supplemental Figure Legends.

Supplemental Figure 1. Impact of CO_2 on Mtb growth *in vitro*. Statistical analysis of Day 9 endpoint data shown in Figure 1C. **A)** WT Mtb exhibits a slight but significant reduction of growth at 5% CO_2 as compared to 0.5% CO_2 at both acidic and neutral pH. **B)** At 0.5% CO_2, the Δ*phoPR* mutant has significantly reduced growth at neutral pH and significantly increased growth at acidic pH. At 5% CO_2, no differences are observed. * P<0.05 in a t-test. Mean ± SD are shown in the bar graph.

Supplemental Figure 2. qRT-PCR confirmation of *canA* and *canB* CRISPRi in WT CDC1551. A) $dCas9_{Sth1}$ knockdown of *canA* target in Mtb. Three sgRNAs targeting *canA* were co-expressed with $dCas9_{Sth1}$ (+ATc). After 6 days of incubation in 7H9 media, total RNA was extracted, and *canA* knockdown was quantified by qRT-PCR. **B)** Two sgRNAs targeting *canB* were co-expressed with $dCas9_{Sth1}$ (+ATc) and *canB* knockdown was quantified by qRT-PCR. **C)** Three sgRNAs targeting *canC* were co-expressed with $dCas9_{Sth1}$ (+ATc). *canC* knockdown was quantified by qRT-PCR but lacked knockdown efficiency. Error bars in all three figures represent the standard deviation of three technical replicates. Significance was determined by two-way ANOVA (Šídák's multiple comparisons test; ****P < 0.0001). Mean ± SD are shown in the bar graph.

Supplemental Figure 3. PCR and qRT-PCR confirmation of *canC* ORBIT knockout and CRISPRi. A) PCR amplification of the 5'(oriE) and 3' (HygC-out1/2) junctions of CDC1551 *ΔcanC*. **B)** PCR analysis of the integration site of the payload plasmid (pKM464). pKM464 is 3082 bp which is consistent with the size of the bands observed in the *canC* deletion mutants compared to WT *canC* which is 2295 bp. **C)** qRT-PCR analysis confirming the *canC* knockout. Total RNA was collected after samples were grown for six days in 7H9 media buffered to pH 7.0. Fold expression was normalized to WT. Error bars represent the standard deviation of three technical replicates. Deletion mutant of *canC* typically exhibited a non-specific primed Ct ~35 cycles compared to WT which had a Ct of ~16 cycles. **D)** qRT-PCR confirmed gene knockdown of *canA*, *canB,*and *canAB* in the *canC* deletion mutation background. Fold expression was normalized to WT Mtb. Error bars represent the standard deviation of three technical replicates. Significance was determined by student's t-test. (*P < 0.05, ***P < 0.001, ****P < 0.0001).

Supplemental Figure 4. Nine day bacterial viability CFUs that correspond to the endpoint data summarized in Figure 2F. BMDMs infected with the CA CRISPRi strains in the CDC1551 Δ*canC* background. All strain treatments were performed in triplicate. Error bars indicate standard deviation.

Supplemental Figure 5. Venn diagram of down-regulated genes 5% CO_2 vs 0.5% CO_2, pH 5.7 compared to up-regulated *phoP::*Tn profile. A) qRT-PCR data demonstrates that *aprA* is induced at 5% CO_2 at pH 5.7 and expression is dependent on *phoPR*. ND= transcript not detected. **B)** Significant gene overlap observed between genes downregulated (Down) (>1.5-fold, q < 0.05) by 5% CO_2 treatment at pH 5.7 and Upregulated (Up) (>1.5-fold, q < 0.05) in the *phoP::*Tn mutant strain at pH 5.7[12]

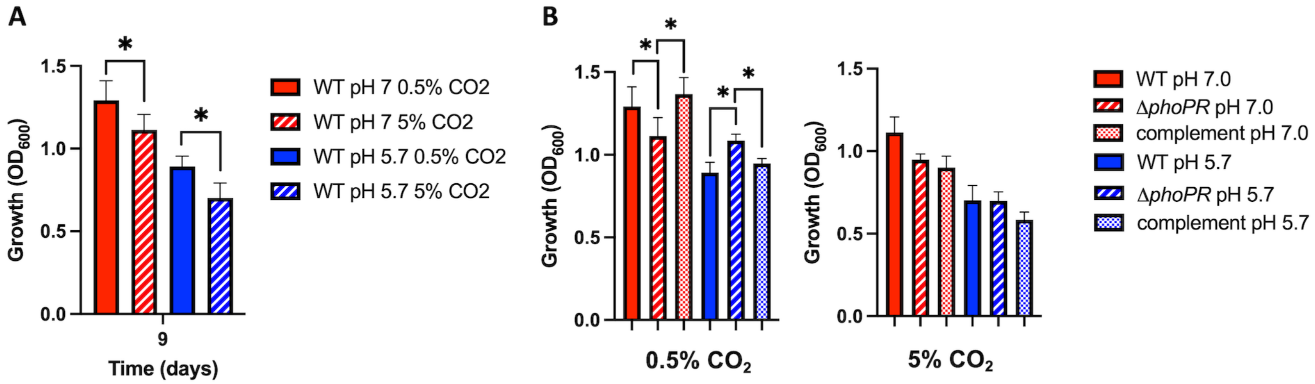

Supplemental Figure 1

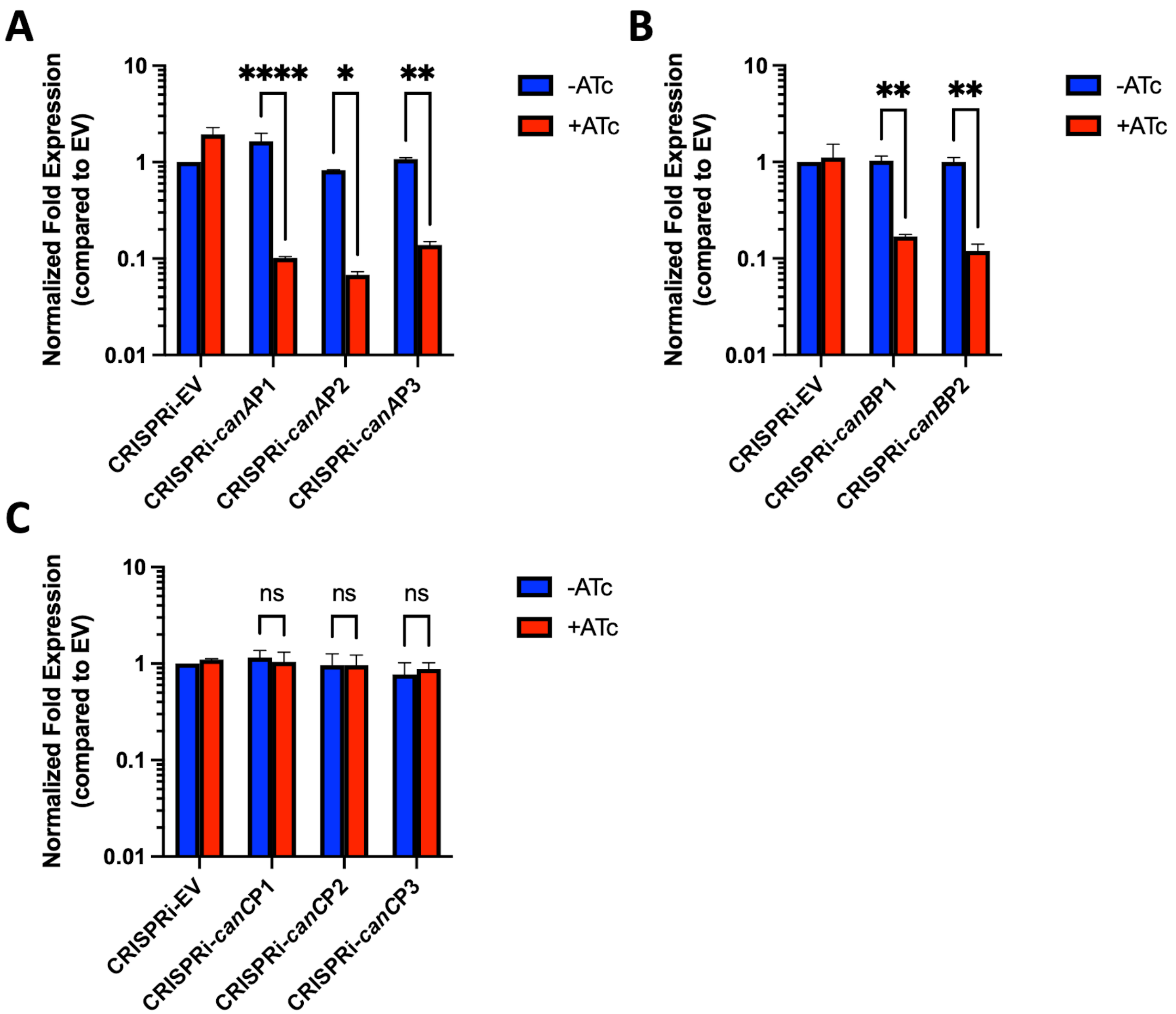

Supplemental Figure 2

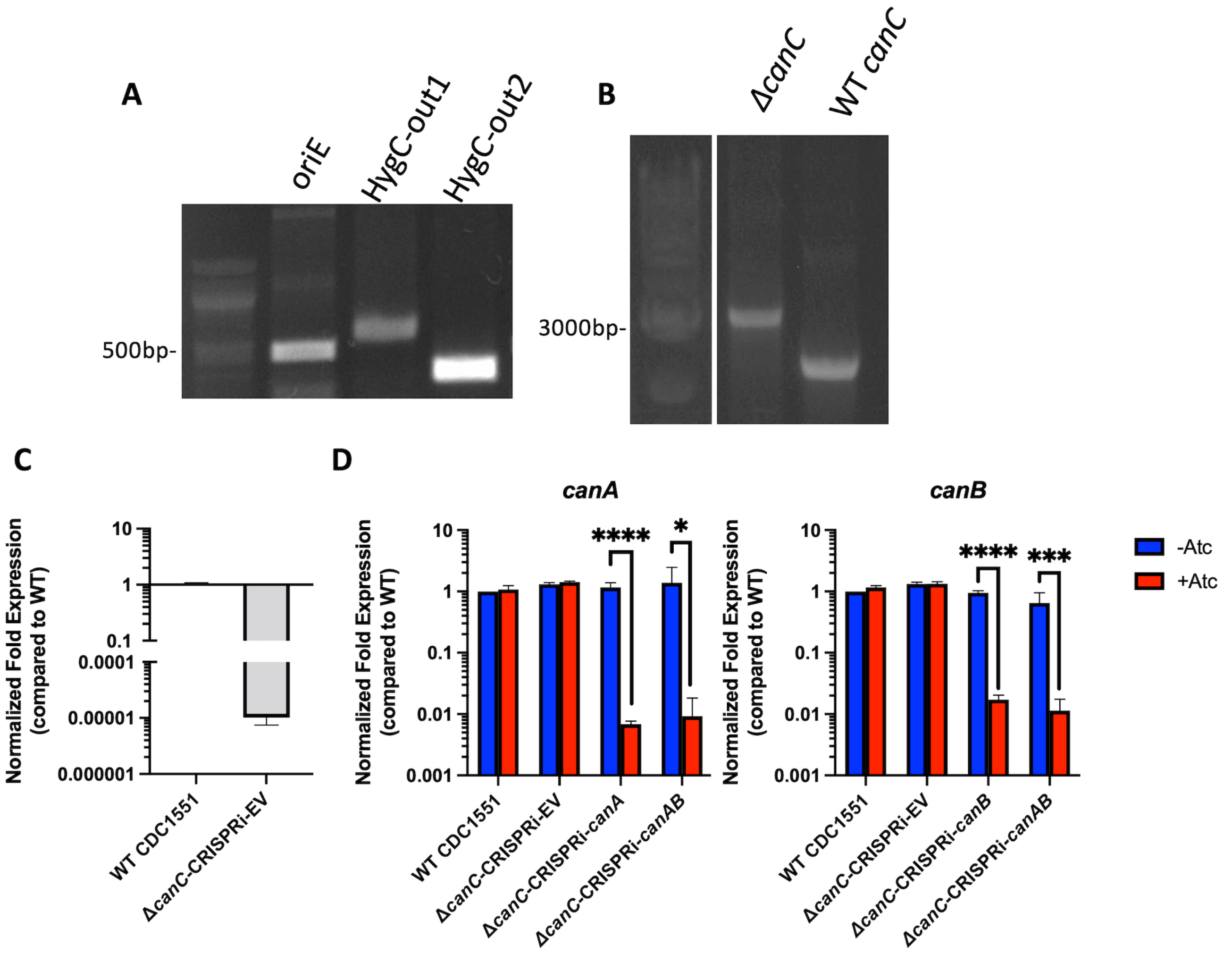

Supplemental Figure 3

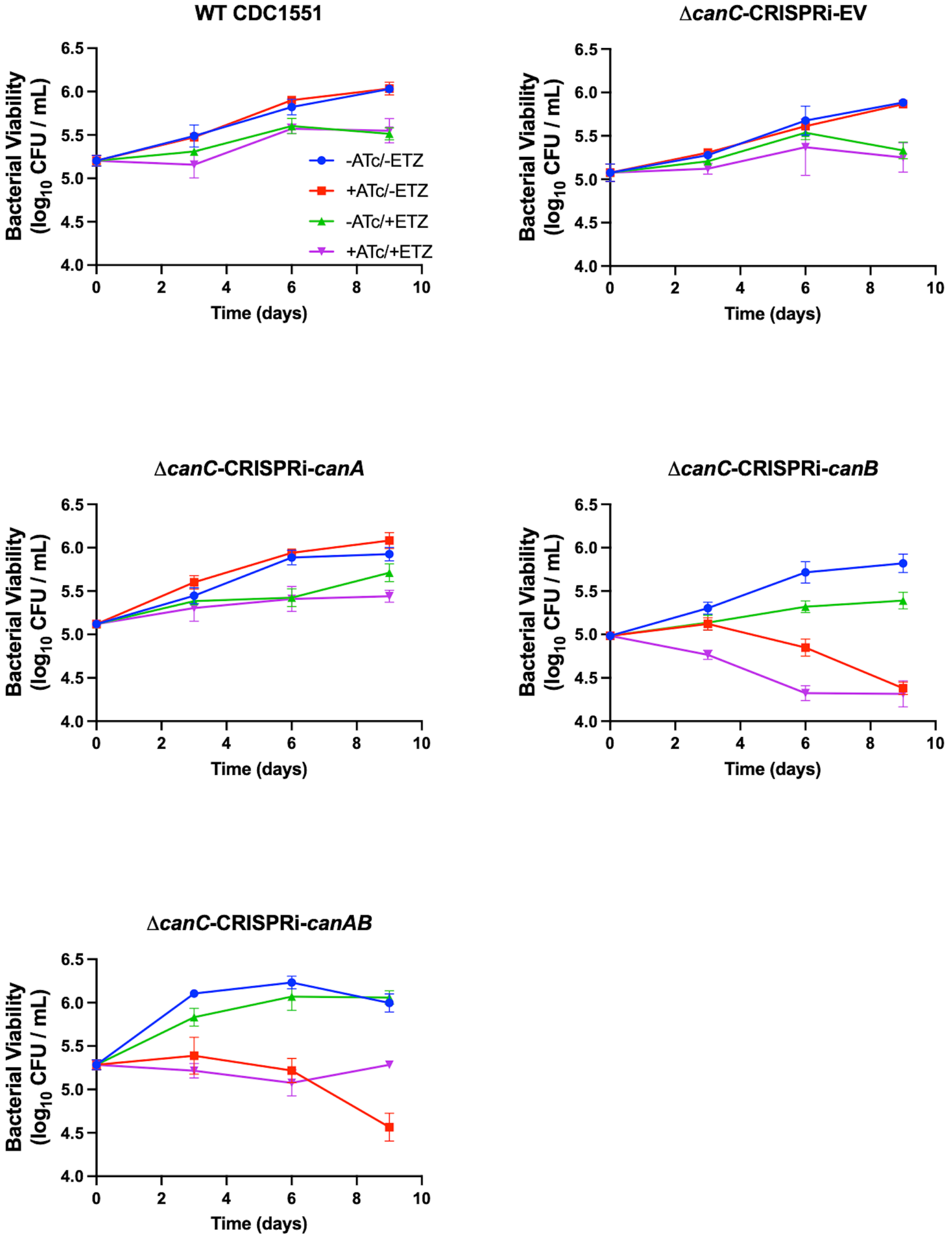

Supplemental Figure 4

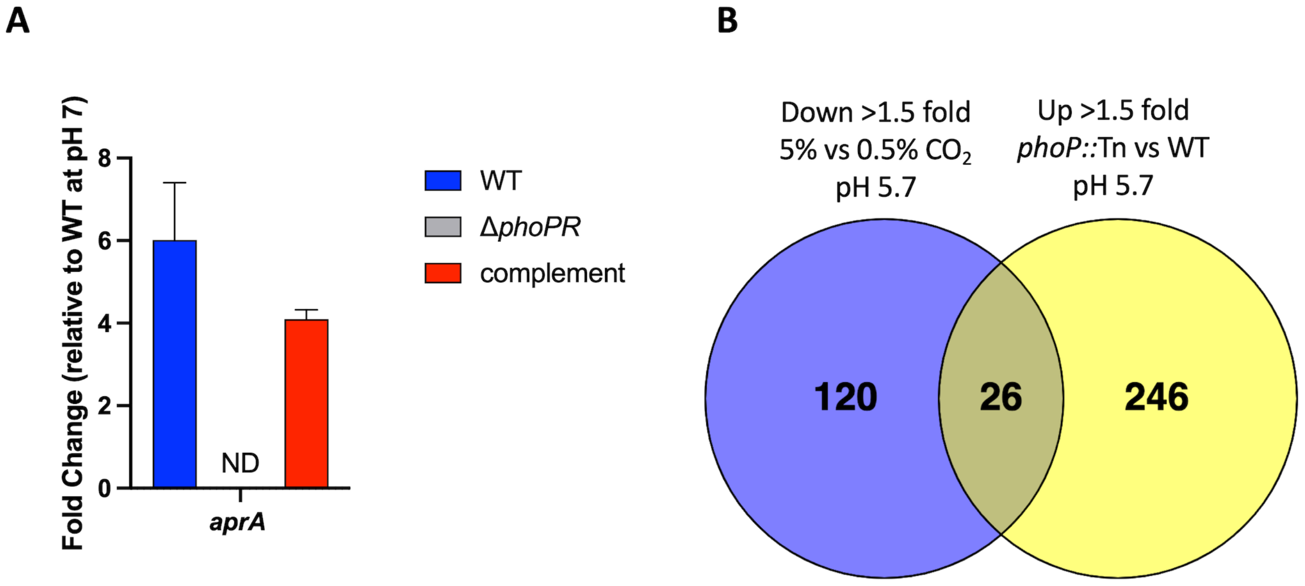

Supplemental Figure 5
